# Adherence to routinely prescribed medications among paediatric sickle cell disease patients in Kumasi, Ghana

**DOI:** 10.4314/gmj.v58i2.2

**Published:** 2024-06

**Authors:** Merlene A Agyekum, Samuel B Nguah, Joseph Attakorah, Gustav K Nettey, Kwaku G Oppong, Vivian Paintsil, Alex O Akoto, Kwame O Buabeng

**Affiliations:** 1 Department of Pharmacy Practice, Faculty of Pharmacy and Pharmaceutical Sciences, Kwame Nkrumah University of Science and Technology, Kumasi, Ghana; 2 Directorate of Child Health, Komfo Anokye Teaching Hospital, Kumasi, Ghana; 3 School of Medicine and Dentistry, Kwame Nkrumah University of Science and Technology, Kumasi, Ghana

**Keywords:** Sickle Cell Disease, Adherence, Ghana

## Abstract

**Objective:**

To measure the adherence to routinely prescribed medications among Sickle Cell Disease (SCD) patients in Kumasi, Ghana

**Design:**

A cohort study involving paediatric SCD patients presenting to the outpatient clinic at the Komfo Anokye Teaching Hospital (KATH).

**Setting:**

The Pediatric SCD Outpatient Clinic of KATH.

**Participants:**

Two hundred SCD patients visiting the outpatient clinic

**Intervention:**

None

**Main Outcome Measure:**

Cost and adherence to Penicillin V, Folic Acid, Hydroxyurea and Vitamins prescribed to SCD patients on routine outpatient visits.

**Results:**

Of the 200 participants, the highest and lowest phenotypes were Hb-SS (162, 80.1%) and Sβ-thal (3, 1.5%), respectively. The adherence rate to routine medications was 13.5% (95%CI: ut9.1% to 19.0%). Patient factors that affect adherence included forgetfulness 98(49%), cost 54(27%), and side effects 28(14%) of medication, and improvement in health 7(3.5%). Hydroxyurea was the costliest to the patients with a median (IQR) cost of GHc 75 (0, 450) or USD12 (0, 73), and the least was folic acid with a median of GHc 6 (0, 42) or USD 1 (0, 7). 44.5% of all medications prescribed were not obtained in full. 83% of those who did not purchase all the medicines attributed this to cost, with 13% indicating they had some at home.

**Conclusion:**

There is low adherence to routine medications used by SCD patients in Kumasi, and this could be because of their relatively high cost. Further studies should be made to assess the non-adherent effects of SCD medications on patients' health.

**Funding:**

This work was funded by the Sickle Pan-African Research Consortium (SPARCO), Kumasi-Ghana site.

## Introduction

Adherence to medications can be defined as the extent to which patients take their medications as prescribed by their healthcare provider. It could also be termed compliance, which is the degree to which a patient conforms to the day-to-day treatment by their provider, taking into consideration the medication's timing, dosage and frequency.^1^ Non-adherence is the opposite and could be defined as the extent to which patients do not take medications as prescribed by their healthcare providers.

Adherence requires that the prescription is obtained promptly and drugs are taken as prescribed in terms of the right dose, dosing interval, duration of therapy, and other specified instructions given by the provider.^2^ In previous studies, it was identified that patient adherence can affect the effectiveness of treatment, its outcome, as well as the quality of life of the patient.^3^

Sickle Cell Disease (SCD) is a genetic disorder that affects millions of people worldwide. The CDC data and statistics analysis confirms about 1 in 13 Black or African American babies is born with a sickle cell trait.^4^ Africa has the highest disease prevalence, with figures suggesting that between 10% and 40% of its entire population may be affected. The incidence of sickle cell trait ranges from 20% to 30% in Cameroon, the Democratic Republic of Congo, Gabon, Ghana, and Nigeria.^5^ In Ghana, the trait prevalence is reported to be 25%, with an SCD prevalence of 2%. A study conducted in the Volta region of Ghana reported the SCD prevalence as 16%.^6^

SCD and its related comorbidities and complications result in high acute healthcare utilisation, with Vaso-occlusive crises (VOC) remaining the primary reasons for SCD patients' emergency room visits and inpatient admissions. Frequent complications during the VOC episodes include infectious diseases, fever, and pulmonary disorders. SCD patients are particularly susceptible to infections, most commonly respiratory tract infections and septicaemia, which can occur as early as six months of age.^7^ This is partly due to splenic dysfunction, which reduces the ability of the immune system to clear circulating antigens.^8^ In addition, abnormalities have been suggested in components of the immune system such as complement, immunoglobulins, leucocyte function and cell-mediated immunity, further disabling the response to infection.^9^ Tissue damage and bone necrosis may also harbour infectious agents.^10^ These abnormalities increase the risk of encapsulated bacterial infections such as pneumococcus and an increase in *Haemophilus influenzae, Neisseria meningitidis, Staphylococcus aureus* and *Escherichia coli* septicaemias. Prevention of these complications includes the use of penicillin prophylaxis started in the newborn period, appropriate immunisations, and blood transfusions for those at risk for stroke.^11^

In the Prophylaxis with Oral Penicillin in Children with Sickle Cell Anaemia (PROPS) study, when infants received prophylactic penicillin between three months and three years of age, pneumococcal infection rates decreased by 84%.^12^ PROPS II evaluated the consequences of discontinuing penicillin prophylaxis at five years of age, and there was no difference in the rates of infection in the penicillin arm compared with the placebo arm.^13^ Based on the PROPS and PROPS II results, children younger than three should receive 125mg of penicillin orally twice daily, and children between three and five should receive 250mg of penicillin orally twice a day. For patients allergic to penicillin, erythromycin ethyl succinate 20mg/kg, divided into two daily doses, can provide adequate prophylaxis. Problems with penicillin prophylaxis include compliance, drug cost, patient tolerance, and resistant strains of micro-organisms. Despite this, SCD patients must adhere to their penicillin prophylaxis to reduce significant infections.^14^

Studies indicate that non-adherence to medications and monitoring are barriers to treatment in SCD.^15^ Factors that can affect adherence in sickle cell disease include the cost of medicines and availability of the medication on the market. This study, therefore, aimed to measure adherence to routine medicines used in the outpatient management of paediatric SCD.

## Methods

### Study site

The study was conducted at the Komfo Anokye Teaching Hospital (KATH), specifically the paediatric sickle cell disease clinic. KATH is in the Kumasi metropolis, the Ashanti Region's regional capital. The Ashanti Region is the second largest of the 16 administrative regions of Ghana, with a population of 5.4 million. It is centrally located in the country and accessible from all corners.^16^

KATH is a tertiary hospital with a bed capacity of 1200 and serves as a major referral centre for the middle and the northern zones of Ghana. The Child Health Directorate of KATH runs specialist outpatient clinics and six inpatient wards. The SCD outpatient clinic is one of the specialist clinics run by the directorate 4 days a week. It's run by four specialist paediatricians, three resident doctors and six nurses. On average, the clinic sees about 500 SCD patients monthly.^17^

Based on the standard operating procedure for managing SCD at the clinic, SCD patients below 3 years and those 3 years and above are scheduled for a visit every 2 and 3 months, respectively. Again, routine laboratory tests such as complete blood counts with reticulocyte counts, kidney function tests, liver function tests, and eye examinations are done. Regarding routine regimen, SCD patients are given folic acid, hematinic and multivitamins, hydroxyurea (Hb-SS genotype and some Hb-SC genotype with severe crisis), and penicillin V (prescribed to all ages).

### Study design

The study employed a prospective cohort design that involved quantitative data collection techniques of SCD patients who presented at the outpatient clinic from October 2021 to January 2022. After this, follow-ups were done to ascertain the number and cost of the prescribed medications purchased.

### Study population

The study population consisted of children with homozygous SCD (Hb-SS), Sickle Cell Beta Thalassaemia (Hb-Sβ-thal), and Sickle Hemoglobin C disease (Hb-SC), proven by HPLC with family studies where necessary, who attended the SCD clinic at KATH.

Participants were children between 1 and 18 years who were free from illnesses necessitating inpatient care at the time of data collection and registered into the Kumasi Sickle Pan African Research Consortium (SPARCo) registry.^18^

### Sample size determination

Based on a pilot study (unpublished) in the same population, we assumed a non-adherence rate of 85%. Using a precision of 5% and a 95% confidence limit, we estimated that approximately 196 patients would be needed for the study.

### Data acquisition

Data was obtained from the Sickle Cell Clinic, patients' folders, interactions with patients or guardians and prescribers, and their reviews of patients' records. This was retrieved using a structured questionnaire from selected patients as part of the study procedure, verified, and uploaded to a protected SPARCO-owned electronic webbased database (RedCap). Patients were systematically sampled from the numbers that visited the clinic routinely. A consent form that included the study's objectives was created and thoroughly explained to patients' caregivers. Caregivers and patients who agreed to participate were made to sign the consent forms. After the consent forms were signed, a designed questionnaire was used to interview participants. Participants who could not provide answers independently had their caregivers do that. After patients visited the care provider, information on the prescription was taken before the patients and their caregivers left the hospital. A medication adherence rating scale (MARS) form was used to determine their medication adherence. This Scale was easy to administer to patients since the questions were straightforward. The adherence scale helped us identify barriers to adherence and when exactly patients took their medications, after which it was compared to the Morisky Medication Adherence Scale.

Phone interviews were conducted to follow up on patients' medication adherence after they left the hospital. This information was used to retrieve the cost of their medications. This interview also helped to ascertain whether medications were bought fully or half-bought. The phone interview followed up when patients were due for review; hence, patients who ran out of medications before review time were determined.

### Data management

The data was entered into a pretested hard-copy questionnaire. Then, a database was created using RedCap version 13.4.10. Afterwards, the data was entered into Red-Cap and cleaned regularly for abnormal values. After data entry, it was exported to R statistical software version 4.2.1 for analysis.

### Statistical analysis

Clinical and demographic characteristics were tabulated and presented as numbers with percentages after stratifying them by SCD phenotype. Stunting, malnourished, and underweight were present when height-for-age, weight-for-height, and weight-for-age z-scores were less than -2 standard deviation as determined using the WHO anthropometric standards. The patient was said to be adherent if he “does not sometimes forget to take them”, “stop taking them when he feels better”, and “do not take the medications only when sick”. The relationship between adherence and the various clinical and demographic features was determined using a chi-squared test or Fisher's Exact test as appropriate. Finally, data on the prescribed medication, cost and quantum purchased were tabulated for each of the four routine medications prescribed for SCD patients.

### Ethical considerations

Ethical clearance was obtained from the Institutional Review Board of KATH, with reference: KATH IRB/AP/083/21, before the commencement of the study. Informed consent was obtained from each participant's guardian voluntarily after clearly explaining the research objectives and other procedures. Participants were anonymised by being given a unique identifier. The data from the questionnaire was stored on a password-protected computer.

## Results

Overall, 203 patients were contacted and screened for the study. Two were not eligible for the study, and one refused to consent. Thus, 200 patients were recruited and included in the final analysis. Of the patients recruited, 32 (16.2%) were stunted, 2(2.4%) were acutely malnourished, and 9 (4.5%) were underweight.

The adherence rate was 13.5% (9.1% to 19.0%). None of the clinical and demographics were significantly associated with adherence to the routine medications **(Table 2)**. Patient factors that affect adherence include forgetfulness 98(49%), medication cost 54(27%), side effects of the medicines 28 (14%), and health improvement 7(3.5%).

Table 3 also outlines that 686 individual drugs were prescribed for all patients. 625 (92.0%) of these were obtained from community pharmacies, with hydroxyurea being the least at 74.6%. The most and least prescribed medications per patient were penicillin V (189, 94.5%) and hydroxyurea (137, 68.5%), respectively. Hydroxyurea was the most expensive, with a median (IQR) cost of GHc 75 (0, 450) or USD12 (0, 73), and the least being folic acid, with a median of GHc 6 (0, 42) or USD 1 (0, 7). Only 372 (55.9%) of the prescribed drugs were bought in full. 72.2% of prescribed folic acid was bought in full, while only 41.8% purchased their vitamins. 229 (83%) of all those who could not purchase all the prescribed medicines attributed this to cost, with 13% indicating they already had some at home. About a quarter of the patients admitted they ran out of their routine medications before the next hospital visit.

**Table 1** outlines these patients' clinical and demographic profiles stratified by SCD phenotype. The highest phenotype seen was Hb-SS (162, 80.1%), with Hb-Sβ-thal being the lowest (3, 1.5%). The ages ranged from less than a year to 17 years, and there were nearly equal numbers of males and females. A hundred and one (50.5%) of the patients were diagnosed through the Sickle Cell Newborn Screening program. Of the patients recruited, 32 (16.2%) were stunted, 2(2.4%) were acutely malnourished, and 9 (4.5%) were underweight.

**Table 1:** Clinical and demographic properties of SCD study patients

		SCD Phenotype		
Characteristic	Overall (N = 200)	SC (N = 35)	SS (N = 162)	Sβ-thal (N = 3)
**Sex**				
Female	99 (49.5)	20 (57.1)	79 (48.8)	0 (0.0)
Male	101 (50.5)	15 (42.9)	83 (51.2)	3 (100.0)
**Age in years**				
< 5 years	49 (28.0)	6 (20.0)	42 (29.6)	1 (33.3)
5 - 10 years	59 (33.7)	11 (36.7)	47 (33.1)	1 (33.3)
> 10 years	67 (38.3)	13 (43.3)	53 (37.3)	1 (33.3)
**Educational Status**				
None	12 (6.0)	0 (0.0)	12 (7.4)	0 (0.0)
Pre-school	71 (35.5)	11 (31.4)	59 (36.4)	1 (33.3)
Primary (class 1 to 6)	76 (38.0)	14 (40.0)	60 (37.0)	2 (66.7)
Junior High	30 (15.0)	7 (20.0)	23 (14.2)	0 (0.0)
Senior High	11 (5.5)	3 (8.6)	8 (4.9)	0 (0.0)
**Mode of SCD diagnosis**				
Newborn Sickle Cell Screening	101 (50.5)	22 (62.9)	77 (47.5)	2 (66.7)
Others	99 (49.5)	13 (37.1)	85 (52.5)	1 (33.3)
**Stunting**	32 (16.2)	5 (15.2)	27 (16.8)	0 (0.0)
**Malnourished**	2 (2.4)	1 (7.7)	1 (1.5)	0 (0.0)
**Underweight**	9 (4.5)	3 (8.6)	6 (3.7)	0 (0.0)

The adherence rate was 13.5% (9.1% to 19.0%). None of the clinical and demographics were significantly associated with adherence to the routine medications **(Table 2)**. Patient factors that affect adherence include forgetfulness 98(49%), medication cost 54(27%), side effects of the medicines 28 (14%), and health improvement 7(3.5%).

**Table 2:** Factors associated with adherence to routine SCD medications by Demographics

		Adherence		
Characteristic	Overall (N = 200)	No (N = 173)	Yes (N = 27)	p-value^2^
**Phenotype**				0.533
SC	35 (17.5)	31 (17.9)	4 (14.8)	
SS	162 (81.0)	140 (80.9)	22 (81.5)	
Sβ-thal	3 (1.5)	2 (1.2)	1 (3.7)	
**Sex**				0.164
Female	99 (49.5)	89 (51.4)	10 (37.0)	
Male	101 (50.5)	84 (48.6)	17 (63.0)	
**Age in years**				0.123
< 5 years	49 (28.0)	39 (25.8)	10 (41.7)	
5 - 10 years	59 (33.7)	50 (33.1)	9 (37.5)	
> 10 years	67 (38.3)	62 (41.1)	5 (20.8)	
**Educational Status**				0.064
None	12 (6.0)	7 (4.0)	5 (18.5)	
Pre-school	71 (35.5)	61 (35.3)	10 (37.0)	
Primary (class 1 to 6)	76 (38.0)	68 (39.3)	8 (29.6)	
Junior High	30 (15.0)	26 (15.0)	4 (14.8)	
Senior High	11 (5.5)	11 (6.4)	0 (0.0)	
**Mode of SCD diagnosis**				0.275
Newborn SCD Screening	101 (50.5)	90 (52.0)	11 (40.7)	
Others	99 (49.5)	83 (48.0)	16 (59.3)	
**Stunting**	32 (16.2)	25 (14.7)	7 (25.9)	0.161
**Malnourished**	2 (2.4)	2 (2.9)	0 (0.0)	>0.999
**Underweight**	9 (4.5)	7 (4.0)	2 (7.4)	0.349

Table 3 also outlines that 686 individual drugs were prescribed for all patients. 625 (92.0%) of these were obtained from community pharmacies, with hydroxyurea being the least at 74.6%. The most and least prescribed medications per patient were penicillin V (189, 94.5%) and hydroxyurea (137, 68.5%), respectively. Hydroxyurea was the most expensive, with a median (IQR) cost of GHc 75 (0, 450) or USD12 (0, 73), and the least being folic acid, with a median of GHc 6 (0, 42) or USD 1 (0, 7). Only 372 (55.9%) of the prescribed drugs were bought in full. 72.2% of prescribed folic acid was bought in full, while only 41.8% purchased their vitamins. 229 (83%) of all those who could not purchase all the prescribed medicines attributed this to cost, with 13% indicating they already had some at home. About a quarter of the patients admitted they ran out of their routine medications before the next hospital visit.

**Table 3:** Medication source, cost and use among SCD study patients

		Medication Prescribed			
Characteristic	Overall (N = 686)	Folic Acid (N = 185)	Hydroxyurea (N = 137)	Pen V (N = 189)	Vitamins (N = 175)
**Outlet drug bought from**					
Community pharmacy	625 (92.0)	175 (95.1)	100 (74.6)	180 (96.3)	170 (97.7)
Hospital Pharmacy	27 (4.0)	5 (2.7)	18 (13.4)	3 (1.6)	1 (0.6)
Hospital Cash Pharmacy	24 (3.5)	4 (2.2)	13 (9.7)	4 (2.1)	3 (1.7)
Others	3 (0.4)	0 (0.0)	3 (2.2)	0 (0.0)	0 (0.0)
**Cost of drug**, *median (IQR)*	18 (0, 450)	6 (0, 42)	75 (0, 450)	12 (0, 70)	18 (5, 200)
**All drugs bought**	372 (55.9)	130 (72.2)	74 (56.1)	97 (53.0)	71 (41.8)
**Reason not all drugs were bought**					
Cost	229 (83.0)	42 (89.4)	52 (94.5)	66 (83.5)	69 (72.6)
Had Some Already	37 (13.4)	5 (10.6)	3 (5.5)	13 (16.5)	16 (16.8)
Complete Part First	10 (3.6)	0 (0.0)	0 (0.0)	0 (0.0)	10 (10.5)
**Run out of medications before clinic**	167 (25.1)	48 (26.7)	35 (26.3)	41 (22.4)	43 (25.3)

## Discussion

SCD and its complications pose a huge burden on patients, guardians, and healthcare workers. Even in a stable state without acute illnesses, the SCD patient is required to be on regular preventive therapy. Despite the well-documented benefits of these routine medications, poor adherence, for any reason, would undoubtedly erode the intended benefits. In this regard, adherence to sickle cell medications is key in its management. In this study, we observed a low adherence rate among SCD patients and determined that none of the social and clinical features of the patients was associated with poor adherence. Furthermore, the study showed a relatively high average cost per drug prescribed, the even higher cost of hydroxyurea and vitamins, the relatively high rate of patients running out of medications before their next visit and the fact that cost was the main reason patients did not purchase all medicines prescribed.

Knowing this information and being able to inform caregivers of the need for medication adherence, this current study aimed to assess the adherence of patients to sickle cell medication since non-adherence leads to an increase in healthcare costs and further complications. The medications assessed were Penicillin V, Folic Acid, Hydroxyurea and Zincovit®.

The study highlighted that many patients do not follow healthcare provider instructions on how medications should be taken, which was evident in the observed low adherence rate. Low adherence could be caused by forgetting to take medications, the unpleasant side effects of these medications, and the cost, which were the main reasons for non-adherence in this context.^19^ It was observed that many caregivers could not afford to buy medications continuously as prescribed by the hospital. Complications mostly seen in SCD patients, such as general body pain, especially, are because of patients not adhering to medications owing to patients not being able to afford their hydroxyurea.

Adherence to medications is key in disease management since non-adherence can lead to complications and an increase in the cost of hospital burdens. Non-adherence also increases mortality and morbidity in the health sector.^20^ For children with sickle cell disease, it is especially necessary for them to be kept on their medications. From the study, a smaller number of children adhered to the medications relative to the number of recruits for the study. Although the effects of non-adherence were not measured in this study, it may still be inferred that children who are non-adherent on their SCD medications often come in with sickle cell crises compared to children who are adherent on medications.

Low adherence in pediatric Ghanaian SCD patients could result in low quality of life, longer hospital stays, decreased health outcomes and overall healthcare costs for these same caregivers.^21^ The factor accounting for nonadherence most in this study was cost. This can be compared to other studies. This was the same as in a study by (Walsh, 2014), which assessed medication adherence among pediatric patients with sickle cell disease. It was concluded in the study that barriers that contributed to adherence included cost, with others, fear of side effects, incorrect dosing, and forgetting.^22^ Also, non-adherence was associated with more Vaso-occlusive crises and hospitalisations. Suggestions are made for a study on the measurements of non-adherence to SCD medications to be carried out in these children for a better argument to be made on adherence to SCD medications in such children.

To limit cost as a barrier to adherence, policymakers, including the government, should be involved in helping reduce the burden of medication cost by ensuring these medications are always made available on health insurance. This will ease the cost burden on patients and their caregivers.

Comparing the cost of SCD medications in Ghana to Nigeria, a study conducted in Nigeria showed that 100 tablets of hydroxyurea pack cost N12,800 (approximately 40 dollars), which is quite expensive for most Nigerians^23^. In contrast, a similar pack in Ghana costs 470 cedis (approximately 39 dollars).^23^ The cost of these medications could be related to the source of the medications, in that, locally manufactured medications are less expensive than medications manufactured outside the country. Hence, local manufacturers are encouraged to take up the course of manufacturing these medications internally for affordable prices for patients and caregivers.

Medications prescribed for patients were obtained from the community pharmacy, hospital dispensary, cash pharmacy, or chemical outlet. The community pharmacy was the most accessible for patients; hence, most medications were purchased from community pharmacies. Some patients gave reasons why purchases from community pharmacies are commonly done. However, medicines like folic acid and penicillin v are available in the hospital dispensary, as follows: too much time is spent taking medications from the dispensary due to the number of patients, and medicines that are picked from the hospital are cheap on the market and hence could be afforded.

Some patients indicated that they only take their medications when they feel sick. Others also added that they stop taking medications when they feel okay, while others stated they stop taking medications when they feel worse on the medications. During the adherence monitoring, some patients felt medications prevented them from getting sick, and hence, they did well to keep taking their medications.

Despite these revealing findings, the study has a couple of limitations. There could have been recall bias in getting information from the participants. There was also a challenge with the instrument for the adherence measurement; therefore, the possibility of being unable to assess other factors.

## Conclusion

The study reports a low rate of adherence to these medications. Given this, studies should also be made to assess the non-adherent effects of SCD medications on the health of patients so healthcare workers can give better counselling to SCD patients. Hydroxyurea is not easily afforded by all. In effect, policymakers should make it a point to help reduce the burden of SCD medications on the Ghanaian Caregiver by making them readily available and accessible in the hospital dispensaries.
